# Total resection via right mini-thoracotomy for left atrial myxoma in juvenile Carney complex: a case report

**DOI:** 10.1186/s44215-024-00173-3

**Published:** 2024-10-29

**Authors:** Kazumasa Matsunaga, Shigeru Ikenaga

**Affiliations:** Department of Cardiovascular Surgery, Tokuyama Central Hospital, 1-1 Kodacho, Shunan, Yamaguchi 745-8522 Japan

**Keywords:** Cardiac tumor, Carney complex, Mini-thoracotomy, Minimally invasive cardiac surgery

## Abstract

**Background:**

Carney complex is a rare syndrome characterized by skin pigmentation, endocrine disorders, and myxomas. It is particularly notorious for its tendency to exhibit aggressive cardiac myxomas. Herein, we present a case of a juvenile female patient diagnosed with Carney complex who underwent a right lateral mini-thoracotomy.

**Case presentation:**

A 13-year-old girl presented with sudden-onset left hemiplegia and dysarthria. Magnetic resonance imaging revealed multiple areas of restricted diffusion. Echocardiography identified a tumor in the left atrium, suspected to be related to Carney complex based on her medical history and physical examination findings. Surgery was performed via right lateral mini-thoracotomy, which minimized the risk of embolism and ensured a cosmetically favorable outcome. The left atrial wall defect was repaired with autologous pericardium. At 3 years postoperatively, follow-up echocardiography indicated no tumor recurrence and normal cardiac function.

**Conclusions:**

Ongoing follow-ups are essential due to the aggressive nature of the Carney complex and its high recurrence rates. Right lateral mini-thoracotomy offers the advantage of avoiding re-sternotomy and minimizing adhesion dissection, making it the optimal choice for this case.

## Background

Carney complex, a rare syndrome characterized by skin pigmentation, endocrine disorders, and myxomas, often exhibits aggressive cardiac myxomas. Cardiac myxomas with Carney complex account for more than half of all causes of death from this syndrome [1, 2]. Although recurrence rates are high, tumor resection is necessary owing to the high risk of sudden death from mitral valve obstruction or tumor embolism. In this report, we present the case of a juvenile female patient diagnosed with Carney complex who underwent a right lateral mini-thoracotomy.

## Case presentation

**Fig. 1:**
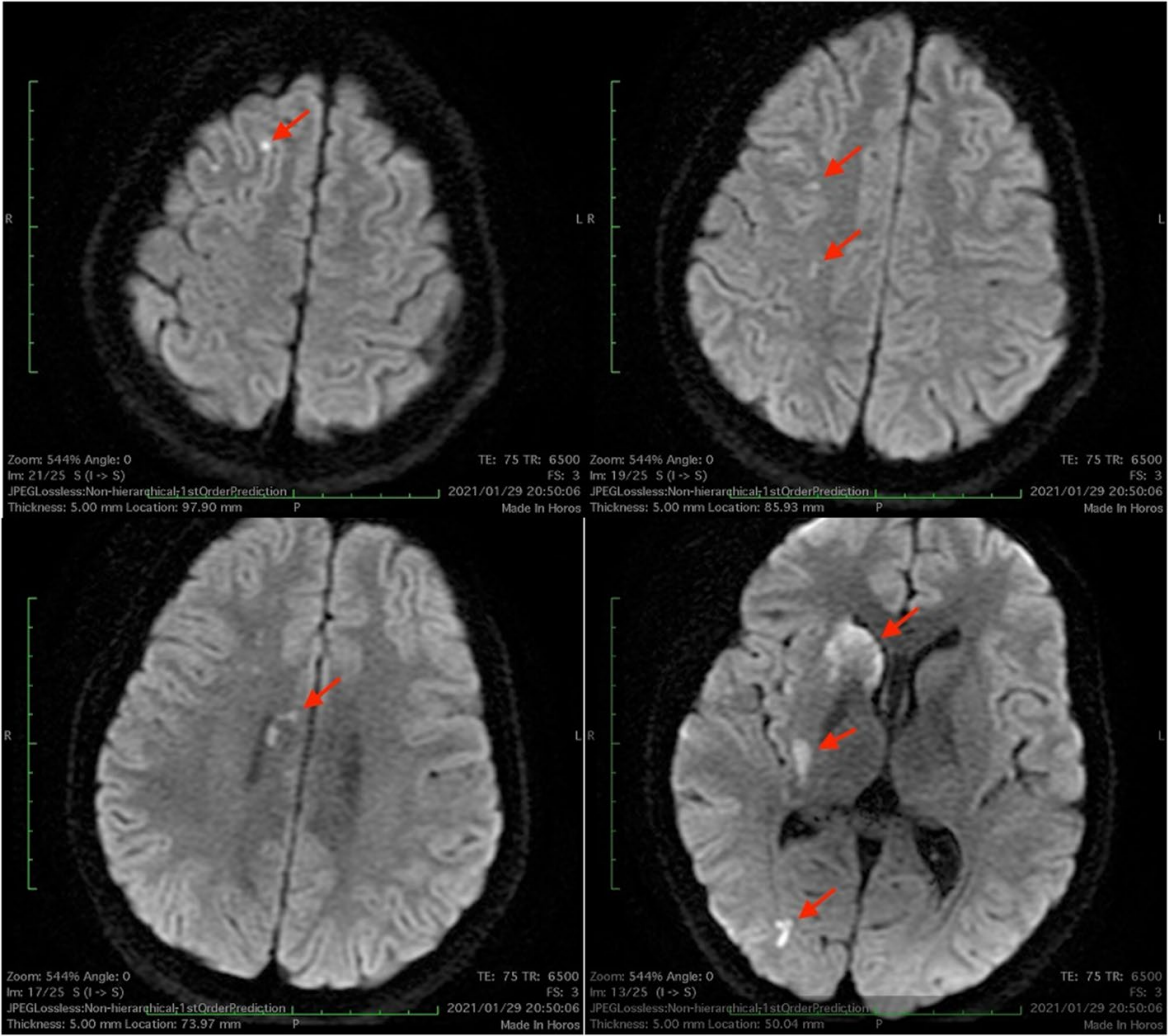
Brain magnetic resonance imaging reveals multiple areas of restricted diffusion (red arrows)

**Fig. 2:**
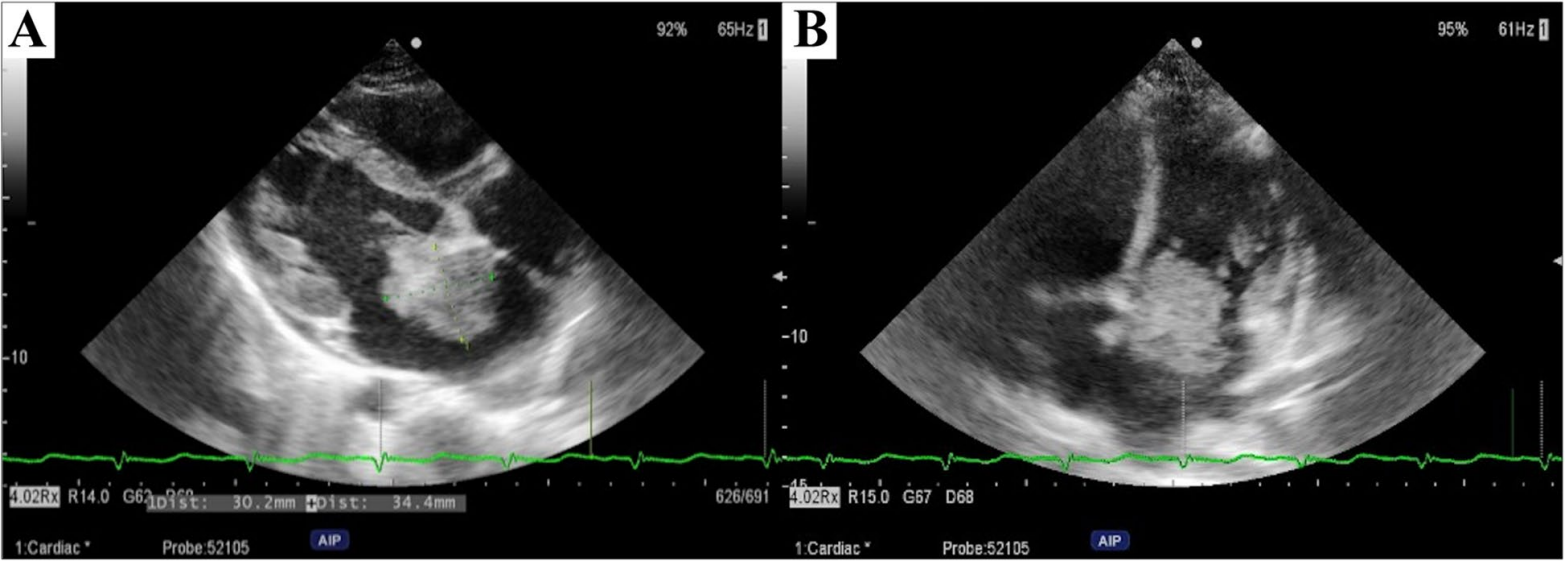
Preoperative transthoracic echocardiographic images. **A** Transthoracic echocardiography shows a tumor arising at the atrial septum in the left atrium, measuring 30 × 34 mm. **B** The cardiac tumor is shown invaginated into the mitral valve during diastole

A 13-year-old girl presented to the emergency department with sudden-onset left hemiplegia and dysarthria. She had a clinical history of the following surgical procedures: accessory nasal sinus tumor excision at the age of 5 years, right maxillary chondromyxoma excision at the age of 8 years, and right ovarian mucinous cystadenoma excision at the age of 11 years. Upon admission, the patient presented with normal vital signs and dotted skin pigmentation scattered around the lip. Magnetic resonance imaging of the brain revealed multiple areas of restricted diffusion involving the right internal capsule, frontal and temporal lobes, and putamen (Fig. 1). Magnetic resonance angiography ruled out stenosis of the brain and carotid vessels, raising suspicion for cardiogenic embolization. Transthoracic echocardiography revealed a tumor measuring 30 × 34 mm in size arising from the atrial septum in the left atrium. The tumor invaginated the mitral valve during diastole, with a mean pressure gradient of 4.6 mmHg (Fig. 2). The laboratory tests revealed a leukocyte count of 6630/mm^3^, a C-reactive protein level of 2.97 mg/dL, an interleukin-6 level of 1.8 pg/mL, and no detected endocrine abnormalities. Carney complex was suspected based on these findings and the patient’s clinical history. We recommended genetic screening; however, the patient’s parent declined.

**Fig. 3:**
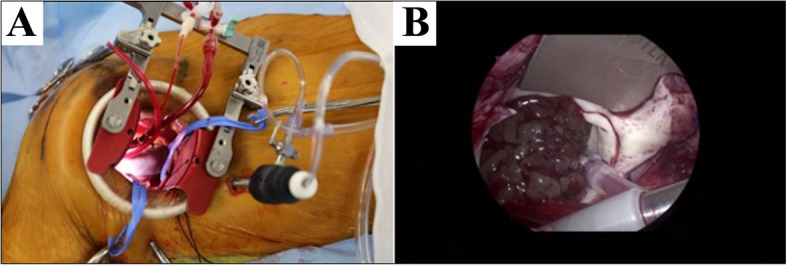
Intraoperative photos. **A** A 6-cm incision is made along the lower margin of the right breast, and a right lateral mini-thoracotomy is performed through the 3rd intercostal space. **B** The giant myxoma extends from the center of the anterior mitral annulus

Surgical resection was performed under general anesthesia, with endotracheal intubation and the patient in the supine position; a 6-cm incision was made along the lower margin of the right breast, and a right lateral mini-thoracotomy was performed through the third intercostal space (Fig. 3A). Cardiac arrest was induced under cardiopulmonary bypass established through arterial cannulation of the right common femoral artery and venous cannulation of the right atrium via the right common femoral vein. The right side of the left atrium was incised to approach the tumor arising from the left atrium. The tumor, a giant myxoma extending from the center of the anterior mitral annulus, was resected along with part of the right atrial wall for complete tumor resection (Fig. 3B). The defect in the left atrial wall, measuring 23 × 10 mm, was reconstructed with an autologous pericardium treated with glutaraldehyde. After rewarming, the patient was successfully weaned off cardiopulmonary bypass with low-dose inotropic support. The total cardiopulmonary bypass time was 156 min, with an aortic cross-clamp time was 95 min. The overall operation time was 252 min.

Histopathological examination confirmed that the tumor was a myxoma, evidenced by the presence of myxomatous stroma with capillary growth and epithelial cells. Carney complex was diagnosed based on the presence of three major criteria: skin pigmentation, a cardiac myxoma, and an osteochondral tumor.

The patient was discharged without any postoperative complications, and the left hemiplegia fully resolved following rehabilitation. Postoperative echocardiography performed at 3 years postoperatively revealed no tumor recurrence and normal cardiac function was confirmed.

## Discussion and conclusions

We achieved complete tumor resection and superior cosmetic outcomes in a 13-year-old girl diagnosed with Carney complex by performing a right lateral mini-thoracotomy. Primary cardiac tumors are rare, with an incidence rate ranging from 0.0017 to 0.28% in autopsy cases [3]. Almost 90% of primary cardiac tumors are benign, and over half are myxomas [1]. Primary myxomas typically affect middle-aged women (aged 30–60 years) and commonly arise in the atrial septum [4]. Complete surgical resection reduces the risk of tumor embolism, with a reported recurrence rate of 3% [1].

Carney complex is a rare autosomal dominant syndrome characterized by skin pigmentation, endocrine disorders, and myxomas. This case was diagnosed as Carney complex due to the presence of skin pigmentation and a history of multiple organ myxomas, despite the patient being only 13 years old. The patient’s family members were healthy and had no related history, suggesting that it was a de novo case. However, the specific gene mutation could not be identified because consent for genetic testing was not obtained. The incidence rate of this syndrome remains to be determined, although over 750 cases have been reported worldwide [2]. Due to the high penetrance, over 95% of individuals develop symptoms by the age of 50 years. Of the reported cases, 70% are hereditary [2]; however, our patient lacked a family history. The characteristics of cardiac myxoma associated with this syndrome include onset at a median age of 20 years, occurrence in any chamber of the heart, familial onset, rapid growth, and a high recurrence rate of 22% after complete surgical resection, in contrast to isolated cardiac myxoma. Cardiac tumors with Carney complex account for more than half of all causes of death from this syndrome [1, 2].

Carney complex is caused by a deficiency in the *PRKAR1A* gene located at the 17q22-24 region. It encodes the regulatory subunit type 1 alpha of protein kinase A, which is involved in intracellular signal transduction, metabolism, and cell cycle progression [2]. Loss of control by the regulatory subunit leads to constant activation of the cAMP-PKA pathway, resulting in tumor development and excessive hormone production. Deletions of components of protein kinase A, including catalytic subunits (PPKACA and PPKACB), have also been reported to be associated with this syndrome [5].

Although recurrence rates are high, tumor resection is necessary due to the high risk of sudden death from mitral valve obstruction or tumor embolism. Preoperative magnetic resonance imaging revealed multiple cerebral infarctions; however, due to the absence of accompanying hemorrhage, emergency surgery was conducted. The approaches considered for tumor resection are median sternotomy and right mini-thoracotomy [6]. In the present case, we opted for the right mini-thoracotomy owing to the absence of tumor invasion and metastasis. Notably, this approach is associated with reduced postoperative pain, fewer infections, and superior cosmetic outcomes [7]. Moreover, as the Carney complex has a high recurrence risk, often necessitating a second surgery, this approach offers the greatest potential advantage of avoiding re-sternotomy and minimizing adhesion dissection [6], making it the optimal choice for this case. This case involved a 13-year-old female with a small physique (height, 155.5 cm; weight, 53.5 kg), and the surgery was safely performed.

In conclusion, right lateral mini-thoracotomy may be a favorable option for Carney complex cases with early-onset invasive tumors and high recurrence rates, offering the advantages of avoiding re-sternotomy, minimizing adhesion dissection, and providing superior cosmetic outcomes.

## Data Availability

Not applicable.
